# Anaesthesia for Fetal Surgeries

**Published:** 2009-10

**Authors:** Kirti N Saxena

**Affiliations:** Professor, Department of Anesthesiology & Intensive care, Maulana Azad Medical college, New Delhi

**Keywords:** Fetal surgery, Fetal interventions

## Abstract

**Summary:**

The concept of the fetus as a patient has evolved from prenatal diagnosis and serial observation of fetuses with anatomical abnormalities.. Surgical intervention is considered when a fetus presents with a congenital lesion that can compromise or disturb vital function or cause severe postnatal morbidity. Hydronephrosis, saccrococcygeal teratoma, hydrocephalus, meningomyelocoele and diaphragmatic hernia are some of the defects that can be diagnosed by imaging and are amenable to intervention.

The combination of underdeveloped organ function and usually life-threatening congenital malformation places the fetus at a considerable risk. Fetal surgery also leads to enhanced surgical and anaesthetic risk in the mother including haemorrhage, infection, airway difficulties and amniotic fluid embolism.

There are 3 basic types of surgical interventions: 1.Ex utero intrapartum treatment(EXIT), 2.Midgestation open procedures, 3.Minimally invasive midgestation procedures. These procedures require many manipulations and monitoring in both the mother and the unborn fetus

## Introduction

The concept of the fetus as a patient has evolved from prenatal diagnosis and serial observation of fetuses with anatomical abnormalities. Rh isoimmunisation provided the first successful example of fetal intervention wherein intravenous blood transfusion was undertaken in a hydropic fetus. Since then several fetal medical interventions like prenatal bone marrow transplantation and prenatal induction of lung maturity have been done^1^. The use of prenatal means of diagnosing anatomical defects is increasing with ultrasound and fetal magnetic resonance imaging. Surgical intervention is considered when a fetus presents with a congenital lesion that can compromise or disturb vital function or cause severe postnatal morbidity. Hydronephrosis, saccrococcygeal teratoma, hydrocephalus, meningomyelocoele and diaphragmatic hernia are some of the defects that can be diagnosed by imaging and are amenable to intervention^2^.

Correcting an anatomical malformation in utero with open fetal surgery jeopardizes the pregnancy and entails potential surgical and anaesthetic risks to the mother as well as the fetus. For this reason fetal surgery remains limited to conditions which if allowed to continue, would irreversibly interfere with fetal organ development but which if alleviated, would allow normal development to proceed. Malformations that qualify for consideration of fetal surgery should satisfy the following prerequisites:

1. Prenatal diagnostic techniques should identify the malformation and exclude other lethal malformations with a high degree of certainty.
2. The defect should have a defined natural history and cause progressive injury to the fetus that is irreversible after delivery.
3. Repair of the defect should be feasible and should reverse or prevent the injury process.
4. Surgical repair must not entail excessive risk to the mother or her future fertility

If new surgical techniques, physiological support systems and tocolytic therapy can reduce fetal and maternal risk to that of elective postnatal surgery, indications for fetal surgery may be liberalized.

## Risks

The combination of underdeveloped organ function and usually life-threatening congenital malformation places the fetus at a considerable risk. Surgery and anaesthesia lead to significant risks to the fetus and can result in fetal death and morbidity. Altered coagulation factors predispose the fetus to bleeding and cause difficulty in achieving surgical haemostasis during fetal surgery. This problem is compounded by the small blood volume of the fetus. Fetal surgery can result in premature labor and birth. Initially surgeries were only performed in cases of impending fetal death.With the advancements in anaesthetic and surgical techniques, the risks have decreased and the indications broadened.

Fetal surgery also leads to enhanced surgical and anaesthetic risk in the mother including haemorrhage, infection, airway difficulties and amniotic fluid embolism. Only ASA class I and II mothers with very sick fetuses are taken up for fetal surgery. Fetal saccrococcygeal tumour leads to the ‘maternal mirror syndrome’, wherein the mother experiences progressive symptoms of preecclampsia due to release of toxins from the placenta.This syndrome is terminated by delivery of the fetus and placenta but not by the excision of the tumour.

## Fetal surgery

### There are 3 basic types of surgical interventions:

#### 1.Ex utero intrapartum treatment(EXIT)^3^

These are also known as OOPS i.e, operation on placental support. These interventions are performed on vaginal delivery or caesarean section. Only a portion of the fetus is delivered and brief procedures such as endotracheal intubation or examination of neck mass done while the fetus is still connected to the placenta through the umbilical cord.Only brief procedures were possible as placental support rarely lasts for more than 10 minutes during routine births. Techniques are being evolved to allow placental support to continue for an hour or longer. It is possible to secure airway in cases ranging from cystic hygroma to complete high airway obstruction syndrome.The following procedures are being done as EXIT procedures:

**Table:** 

Disease	Procedure
• Congenital Diaphragmatic at Hernia	Removal of tracheal bal loon inserted 22–27 weeks with midgestation procedure.ECMO cannulation for severe pulmonary hypoplasia
• Congenital high airway obstruction Syndrome(CHAOS)	Perform tracheostomy
• Giant cervical neck mass	Resection of mass
• Anticipated difficult intubation	Laryngoscopy, bronchoscopy, tracheostomy

#### 2.Midgestation open procedures

Recognition of foetal defect in early pregnancy allows intervention in midgestation to prevent irreversible damage or development of secondary disease.Hysterotomy is required to access the foetus who is returned to the uterus after completion of surgery for the rest of the gestation. Fetal surgery is performed through a low transverse abdominal incision. Placental location is determined by ultrasonography and a wide uterine incision is given by a specially designed absorbable stapler for performing bloodless hysterotomy. The fetal part is exteriorized for surgery and after completion of the surgery the fetus is placed back into the uterus which is closed.

The fetus continues to grow for the rest of the gestation with reversal of the disease process that prompted the fetal intervention. Example is repair of meningomyelocoele at 22 weeks of gestation to prevent damage to central nervous system tissues due to prolonged exposure to amniotic fluid. The sequelae of bladder and bowel dysfunction and clubfeet may be prevented. The indications for open midgestation fetal surgery are:

**Table:** 

Disease	Procedure
• Meningomyelocoele	Surgical repair of meningomyelocoele
• Saccrococcygeal teratoma	Surgical resection of teratoma
• Intrathoracic masses	Resection of mass
• Congenital DiaphragmaticHernia	Temporary tracheal occlusion with intrathoracic liver
• Congenital Cystic Adenoid Malformation	Lobectomy, pneumonectomy

#### 3.Minimally invasive midgestation procedures^4^

Because the uterus is a fluid filled organ, small endoscopes allow excellent visualization of fetal and placental structures as long as uterine distention is maintained with irrigating fluid.These are basically of 2 types:

**1.Fetendo procedures-**Aberrant placental vessels providing imbalance of blood flow to twins can be identified and ligated in this way to prevent fetal death due to twin-twin transfusion syndrome.Other surgeries possible with this technique are radiofrequency ablation or coagulation of non viable twin's umbilical cord in twin reversed arterial perfusion and division of amniotic bands in amniotic band syndrome. Using fetoscopy, these aberrant vessels and bands can be identified and coagulated. Fetal procedures, such as fetal cystoscopy with laser ablation of posterior urethral valves, are also now technically possible and are being undertaken.

**2. FIGS-IT-** This is a term used for fetal image guided surgery for intervention or therapy, and describes the method of manipulating the fetus without either an incision in the uterus or an endoscopic view inside the uterus. The manipulation is done entirely under realtime cross-sectional view provided by the sonogram. This is the same sonogram as is used for diagnostic purposes, but in this case is used to guide instruments.

Like Fetendo, it can be done either through the mother's skin or, in some cases, with a small opening in the mother's abdomen. It can often be done under a regional anaesthesia like an epidural or a spinal, or even under local anaesthesia. This is the least invasive of the fetal access techniques and, thus, causes the least problem for mother in terms of hospitalization and discomfort.

## Anaesthetic goals of fetal surgery

### Maternal anaesthetic considerations

Anaesthetic plan must accommodate all the physiological changes of pregnancy. Pregnancy affects maternal pulmonary and cardiovascular function. There is increased demand of oxygen so adequate precautions should be taken to prevent hypoxaemia and aspiration. Decreases in capillary oncotic pressure and increases in capillary permeability increase the risk of pulmonary edema specially when magnesium sulphate is used for tocolysis.Left uterine displacement to prevent aortocaval compression must be done.Doses of anaesthetic agents should take the increased sensitivity of the parturient into consideration.

### Fetal anaesthetic considerations

Fisk et al^5^ demonstrated increased cortisol, beta endorphins, and the “central sparing response” in a 23 week old human fetus needled in the hepatic (innervated) vein. 10mcg.kg^−1^ fentanyl suppressed the betaendorphin and cortisol responses but not the central sparing response to this noxious stimulus. For the first time it was demonstrated that the human fetal stress response was attenuated by the administration of a narcotic. Long-term effects of fetal stress have also been described. Independent groups have implicated fetal stress to exaggerated pain responses in eight week-old infants and have also implicated the fetal stress response as a contributor to pre-term labor.

There is need for fetal anaesthesia in contrast to caesarean section where a vigorous baby is desirable on delivery. Placental transfer of of anaesthetic agents to the fetus is a desirable effect of maternal anaesthesia. The fetus requires less muscle relaxant and anaesthetic agent.Although inhaled anaesthetics, rapidly cross the placenta,fetal levels remain below maternal levels for a prolonged period of time.

Less cardiac contractile tissue leads to depression from volatile anaesthetic.^6^ Anaesthetic-induced decreases in contractility combined with fetal surgical manipulations can result in hypotension, bradycardia, and eventual cardiac collapse. Because of low circulating blood volume and rate dependent cardiac output, surgical blood loss is poorly tolerated so trigger for transfusion is low.Thin fetal skin predisposes fetus to hypothermia so active warming is required during exposure from uterus. Hypothermia can be minimized by limiting fetal surgical time and use of warm irrigating fluids.

Maintenance of uteroplacental circulation is vital for successful outcome of the procedure. Fetal oxygenation is dependent on both blood oxygenation and placental blood flow. Uterine tone increases during contractions and this increases vascular resistance.Since uteroplacental flow is influenced by vascular resistance therefore uterus must remain relaxed. Kinking of umbilical cord must be avoided and corrected if this has occurred during changing position of the fetus to facilitate blood flow. Increase of maternal pH and hypocapnia result in reduced umbilical blood flow and fetal hypoxia.

### Preoperative preparation^3^

Preanaesthetic checkup is done to evaluate the mother with special emphasis on maternal and family history of anaesthetic problems, airway evaluation and concurrent medical problems.The placental location and fetal cardiovascular function are evaluated by ultrasonography, echocardiography, magnetic resonance imaging.

The patient is admitted on the day of surgery after being nil orally. The operating room is warmed to 80°F and type specific packed red cells for the mother and O-negative packed red cells for the fetus are made available. Monitors include two pulse oximeters, an arterial pressure transducer and cardiac monitor for the mother sodium bicitrate is given orally and metoclopramide given intravenously as prophylaxis for aspiration. An indomethacin suppository is administered for postoperative tocolysis in midgestation procedures.

The fetal care team consists of obstetricians, pediatricians, surgeons specializing in fetal surgery and anaesthesiologists. Intensive care facilities for both mother and neonate are available.

### Fetal monitoring^6^

For procedures where the fetal extremity is accessible, a pulse oximeter probe is placed on the limb and wrapped with foil to decrease interference with ambient light. Normal fetal arterial saturation is 60–70% and values above 40% during surgery represent adequate saturation.

Echocardiography is used to monitor fetal heart rate and stroke volume. Fetal arterial or venous blood gas samples may be obtained by the surgeons percutaneously or through umbilical or central vessel puncture. Warmed, fresh O-negative blood can be administered to the fetus to correct anaemia.

## Anaesthetic plan for Fetal Surgery Procedures

### Ex Utero Intrapartum Treatment Procedures

EXIT procedures require maternal laparotomy and hysterotomy while maintaining uteroplacental circulation. General anaesthesia is preferable as it accomplishes both maternal and fetal anaesthesia.^7^

Adequate preoxygenation is followed by rapid sequence induction. Desirable uterine atony can be maintained with high concentrations of volatile anaesthetics.However, this leads to maternal hypotension which is treated by intravenous fluids and sympathomimetics to maintain mean arterial pressure to within 20% of the baseline to preserve uteroplacental blood flow.

Following uterine incision, the operative site of the fetus is delivered through the hysterotomy. Care is taken to prevent kinking of the umbilical cord.Fetal monitoring with pulse oximetry and echocardiography are undertaken. Before start of the intervention, fetal anaesthesia is supplemented by intramuscular or intravenous injection of vecuronium and fentanyl. If blood loss is high then the surgeon can perform intravenous cannulation of the fetus to give drugs and fluids.Good fetal anaesthesia relieves fetal stress and ensures good operating conditions.

Endotracheal intubation may be undertaken in the fetus with severe cardiac defects to prevent hypoxaemia at birth. Placement of an LMA can be done if the fetus has an airway problem and tracheostomy performed following delivery.

After completion of the procedure the infant is delivered, the umbilical cord divided and the baby placed in the care of a neonatologist. Following this the uterine atony is reversed by reducing the concentration of volatile anaesthetic and giving methergin. Adequate replacement of fluids must be done since EXIT procedures are more prone to blood loss than caesarean section. An epidural catheter may be placed for postoperative analgesia in the mother.

After surgery there are two patients to be cared for, and a second operating room should be available in case further surgery is needed in the neonate.

### Midgestation Open fetal Surgery

This is similar to the EXIT procedures as far as the exposure of the fetus is concerned but is done in the second trimester.Since the fetus is returned to the uterus after completion of the intervention, the parturient requires tocolysis and fluid restriction.

The mother is induced by rapid sequence technique. An arterial catheter is placed before increasing the concentration of volatile anaesthetic upto two and a half MACs. Fetal monitoring is done with pulse oximetry and echocardiography. Fetal analgesia may be supplemented with intravenous or intramuscular medication. After completion of the intervention the fetus is returned to the uterus The amniotic fluid is replaced by warmed Ringer's solution containing antibiotics and the uterus closed.

Aggressive tocolysis is initiated after conclusion of the fetal procedure with an intravenous infusion of magnesium sulphate to prevent reflex uterine contractions. These can result in reduced uteroplacental blood flow and result in premature labor. Magnesium sulphate can increase the risk of pulmonary edema which can be managed by the use of adrenergic agents. Magnesium sulphate also increases sensitivity to muscle relaxants hence neuromuscular monitoring should be done.

In the postoperative period, the mother is maternal-fetal intensive care unit. Uterine activity and fetal heart rate are continuously monito red by tocodynamometry. Use of an epidural catheter reduces the maternal stress response and incidence of early preterm labor^8^.

### Minimally Invasive Surgery

Most procedures can be done with large bore needle under ultrasound imaging or a 5-mm trocar. The fetal surgery trocar has a camera and a port for a laser fibre to coagulate placental vessels in twin-twin transfusion syndrome.In fetoscopy, normal saline irrigation is used inside the uterus. The maternal incision is small and such procedures can be performed under local anaesthesia with infiltration of both skin and peritoneum. Epidural, spinal or combined spinal-epidural anaesthesia can be used^9^

Intravenous sedation should be given for maternal anxiolysis for fetoscopy because of the emotional nature of surgery and the awkward position of the mother. Midazolam, fentanyl, remifentanil or propofol infusion may be used but deep sedation should be avoided to prevent aspiration. The uterus is irrigated with normal saline in this procedure and this can be absorbed. This fluid can also enter the peritoneal cavity through the fallopian tubes from where it can be absorbed. This can lead to pulmonary edema as the patient is also receiving tocolytics. This can be treated with diuretics and may be anticipated when the volume of irrigating fluid is much higher than the returning fluid. Most fetoscopy procedures involve manipulation of placenta and umbilical cord and there is no fetal incision therefore fetal anesthesia is not required.^10^

### The Future

Fetal surgery is a new field that is expanding rapidly.Currently it is being done only in few centers in the world. Anaesthetists are an important part of the team and specialized training is required for fetal anaesthesia. This is a rapidly evolving field and some controversies still need to be resolved. Standardised assessment tools and blood microsampling techniques for the fetus need to be developed to allow further development of clinical protocols. Questions regarding fetal stress and optimal drug dosing in the fetus remain open to speculation until these techniques evolve to answer our questions. In line with the trend in the adult, neonatal minimally invasive congenital heart surgery has become an important area of interest.^11^
